# Knee joint sagittal plane movement in cerebral palsy: a comparative study of 2-dimensional markerless video and 3-dimensional gait analysis

**DOI:** 10.1080/17453674.2018.1525195

**Published:** 2018-12-18

**Authors:** Evelina Pantzar-Castilla, Andrea Cereatti, Giulio Figari, Nicolò Valeri, Gabriele Paolini, Ugo Della Croce, Anders Magnuson, Jacques Riad

**Affiliations:** 1 Department of Orthopedics, Örebro University Hospital, Örebro, Sweden;; 2 Department of Biomedical Sciences, University of Sassari, Sassari, Italy;; 3 Department of Electronics and Telecommunications, Politecnico di Torino, Torino, Italy;; 4 GPEM srl, Alghero, Italy;; 5 Clinical Epidemiology and Biostatistics, School of Medical Sciences, Örebro University, Örebro, Sweden;; 6 Department of Orthopedics, Skaraborg Hospital Skövde and Mölndal Hospital Sahlgrenska, Gothenburg, Sweden

## Abstract

Background and purpose — Gait analysis is indicated in children with cerebral palsy (CP) to identify and quantify gait deviations. One particularly difficult-to-treat deviation, crouch gait, can progress in adolescence and ultimately limit the ability to ambulate. An objective quantitative assessment is essential to early identify progressive gait impairments in children with CP. 3-dimensional gait analysis (3D GA) is considered the gold standard, although it is expensive, seldom available, and unnecessarily detailed for screening and follow-up. Simple video assessments are time-consuming when processed manually, but more convenient if used in conjunction with video processing algorithms; this has yet been validated in CP. We validate a 2-dimensional markerless (2D ML) assessment of knee joint flexion/extension angles of the gait cycle in children and young adults with CP.

Patients and methods — 18 individuals, mean age 15 years (6.5–28), participated. 11 had bilateral, 3 unilateral, 3 dyskinetic, and 1 ataxic CP. In the Gross Motor Function Classification System, 6 were at level I, 11 at level II, and 1 at level III. We compared 2D ML, using a single video camera with computer processing, and 3D GA.

Results — The 2D ML method overestimated the knee flexion/extension angle values by 3.3 to 7.0 degrees compared with 3D GA. The reliability within 2D ML and 3D GA was mostly good to excellent.

Interpretation — Despite overestimating, 2D ML is a reliable and convenient tool to assess knee angles and, more importantly, to detect changes over time within a follow-up program in ambulatory children with CP.

Gait analysis is indicated in children with cerebral palsy (CP) to identify gait deviations, evaluate treatment, and follow the natural history (Bell et al. 2002). One of the most critical and frequent gait deviations in bilateral CP is crouch gait, defined as the inability to fully extend the knee in stance (Gage et al. 1996, Rodda and Graham 2001, Miller 2005). Even if crouch gait is tolerated in the young child, in adolescence with growth, it may progress and ultimately limit the ability to ambulate (Opheim et al. 2013). Several gait variables are relevant in crouch gait (Perry 1992, Benedetti et al. 1998, Sangeux and Armand 2015) (Figure 1). Even if there is treatment for crouch gait the challenge is to identify progressive gait impairments in children with CP early and choose effective treatment (DeLuca 1991, Gage et al. 1996, Rodda et al. 2004, Narayanan 2007). Hence, a systematic quantitative description of gait function is essential (Perry 1992, Benedetti et al. 1998, Sangeux and Armand 2015).

**Figure 1.**
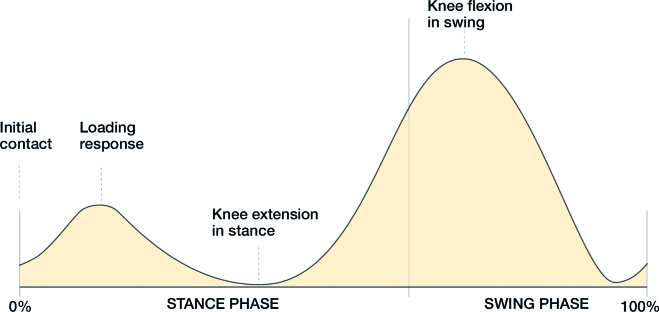
The 4 knee-flexion and extension variables selected for the analysis (stance and swing phase in percentage of the gait cycle on the X-axis). 1: Knee flexion at initial contact (0% of the gait cycle) important for step length, which can be limited by hamstring spasticity and/or short hamstring muscles; 2: maximum knee flexion at loading response (0–40% of the gait cycle) manages force absorption; 3: minimum knee flexion in stance (25–75% of the gait cycle) describes degree of crouch; 4: and maximum knee flexion in swing (50–100% of the gait cycle) contributes to foot clearance.

Marker-based 3-dimensional gait analysis (3D GA) provides a dynamic assessment of gait and is considered the gold standard to quantify lower limb movements, however seldom available since it is expensive and requires a dedicated space (Eastlack et al. 1991, Perry 1992, Mackey et al. 2003). In addition, for screening purposes and for follow-up, the level of detail provided by 3D GA might be unnecessary; knee-joint sagittal angles may suffice as a valid and useful preliminary assessment (Maathuis et al. 2005, Kawamura et al. 2007, Narayanan 2007). To quantify and partially reduce examiner subjectivity, visual scoring systems have been proposed (Narayanan 2007). The Edinburgh Visual Gait Score was specifically developed for assessing gait in CP and has proven to have good reliability and validity (Read et al. 2003, Rathinam et al. 2014, Del Pilar Duque Orozco et al. 2016). However, joint angles, used to score gait, require manual identification of specific anatomical landmarks on multiple videotaped sequences. Clearly, this operation can be quite time-consuming and heavily relies on the operator’s skills and experience (Read et al. 2003, Ong et al. 2008, Gupta and Raja 2012, Del Pilar Duque Orozco et al. 2016). Some of the limitations associated with visual video assessments can be overcome by using automatic video processing (Ugbolue et al. 2013, Castelli et al. 2015, Saner et al. 2017). However, most of the 2-dimensional markerless (2D ML) gait analysis methods proposed in the literature have been applied and validated only on healthy individuals (Surer et al. 2011, Sandau et al. 2014, Castelli et al. 2015, Saner et al. 2017); therefore, the results cannot be generalized to populations with gait disorders, as deviations from typical gait patterns might interfere with the performance and feasibility of the method. To address the above-mentioned clinical requirements, we developed a 2D ML method which takes advantage of a single-color red–green–blue (RGB) camera combined with a depth infrared sensor (RGB-D). The proposed method is an improvement of a recent markerless method developed and validated on healthy adults (Castelli et al. 2015, Saner et al. 2017).

We evaluated the validity of this newly developed 2D ML gait analysis method compared with 3D GA for the estimation of knee flexion and extension angles during gait in children and young adults with CP.

## Patients and methods

### Population

From the medical records in Örebro and Skaraborg, individuals with CP were identified. Those eligible for inclusion were sent a letter with information and invitation to participate. Inclusion criteria were CP and GMFSCS level I–III. 23 were willing to participate, and visited the Skaraborg Gait Analysis Laboratory, Skövde. 3 participants were not able to perform the gait analysis due to cognitive and visual impairments, and 2 were excluded for technical reasons. Gait data were collected for 18 participants, mean age 15 years (6.5–28), 4 females and 14 males. 11 participants had bilateral CP, 3 had unilateral CP, 3 had dyskinetic CP, and 1 had ataxic CP. 6 were classified as GMFCS level I, 11 were at level II, and 1 was at level III.

### Data collection

The participants walked at a self-selected speed on a 7-meter walkway wearing a T-shirt, underwear, and colored ankle socks. The RGB-D camera was positioned laterally to the walkway, and a homogeneous green background was rigged. The image coordinate system of the video camera was aligned to the sagittal plane. The data acquisition and synchronization was performed simultaneously for 2D ML and 3D GA. 5 gait trials per participant and side were recorded.

#### 2-dimensional markerless gait analysis (2D ML)

**Figure 2.**
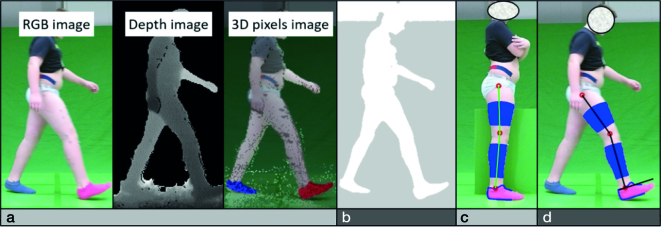
Procedures needed for the estimation of the knee flexion and extension angles with the 2-dimensional markerless video system: image pre-processing calibration (a), segmentation (b), participant-specific multi-segmental model (c), and joint-center tracking (d).

An RGB-D system was used (Kinect 2 for Xbox One [Microsoft Corp, Redmond, WA, USA]), RGB images with resolution 1920 × 1080 pixels at 30 frames/second and depth image of 512 × 424 pixels at 30 frames/second. The estimation of knee flexion and extension angles required the implementation of the following steps (see Appendix in Supplementary data for a detailed description):

1. Image pre-processing calibration: includes optical distortion correction and allows RGB and depth data to obtain a matrix of colored 3D pixels for each frame (Figure 2a).
2. Segmentation: subtraction to separate the participant from the background (Figure 2b).
3. Participant-specific multi-segmental model: a lower-limb kinematic model composed of the foot, shank, and thigh segments connected by cylindrical hinges. To compensate for real and apparent deformations of the body segments, a multiple anatomical landmark calibration was implemented (Figure 2c).
4. Joint center tracking: trajectories of ankle, knee, and hip joints centers were estimated during the gait cycle by minimizing the distance between the measured and modelled body-segment points (Figure 2d). Depth data were exploited to handle segments overlapping during walking.
5. Flexion and extension knee joint angle: orientation of the tibia and femur were determined from joint center trajectories and expressed as percentage of the gait cycle.

#### 3-dimensional marker-based gait analysis (3D GA)

3D GA images were captured at 100 frames/second using a 12-camera stereo-photogrammetric system (Oqus 400 Qualisys medical AB, Gothenburg, Sweden). 38 retro-reflective spherical markers were attached to the participants according to the modified Helen-Heyes model. Calculation of joint angles was performed using the Visual 3D software (C Motion Inc., USA).

### Knee joint gait variables

For each participant and trial, the knee flexion and extension angles were estimated for 2D ML and 3D GA (see Figure 1). Time synchronization between 2D ML and 3D GA curves was performed. The following knee variables were extracted from the 2D ML and 3D GA curves:

- Knee flexion at initial contact (0% of the gait cycle), is important to achieve an effective “prepositioning of the foot” and “adequate step length.” These are two prerequisites of normal gait, depending on the ability to extend the knee before initial contact in late swing phase (Perry 1992, Benedetti et al. 1998).
- Maximum knee flexion at loading response (0–40% of the gait cycle), is controlled mainly by the quadriceps muscle with eccentric- and isometric muscle contraction to stabilize the knee, and for shock absorption (Sangeux and Armand, 2015).
- Minimum knee flexion in stance (25–75% of the gait cycle), provides a measure of crouch gait and is influenced by fixed knee flexion, hamstring spasticity and/or short hamstring muscles, and foot stability, affecting a third prerequisite of normal gait: “stability in stance” (Perry 1992).
- Maximum knee flexion in swing (50–100% of the gait cycle), may be limited and delayed by prolonged rectus femoris muscle activity in swing, influencing both “adequate step length” and “clearance in swing” (Perry 1992). The decreased knee flexion in swing is often presented as toe-drag, not to be confused with limited ankle dorsiflexion in swing (Sangeux and Armand 2015).

### Stastistics

For each variable, the mean value of 3 trials was calculated for each participant and side. Normal distribution was assessed. Bland–Altman plots with 95% limits of agreements (LoA) were used to compare 2D ML and 3D GA methods for the selected variables. In the Bland–Altman plot, the Spearman correlation coefficient was used to further evaluate if differences between methods were correlated to the mean values of knee flexion. Unpaired t-test with 95% confidence intervals (CI) was used to quantify potential systematic mean differences between the 2D ML and 3D GA methods. For each variable, method reliability (2D ML and 3D GA) was evaluated with intraclass correlation (ICC) based on absolute agreement and 2-way random effects. Based on the CI of the ICC estimate, values less than 0.5 indicate poor reliability, values between 0.5 and 0.75 indicate moderate reliability, values between 0.75 and 0.9 indicate good reliability, and values greater than 0.90 indicate excellent reliability (Koo and Li 2016). A p-value less than 0.05 were regarded as statistically significant, and the Bonferroni method was used to correct for multiple testing. All statistical analyses were performed in SPSS for Windows, version 22 (IBM Corp, Armonk, NY, USA).

### Ethics, funding, and potential conflict of interest

The study was approved by the regional ethical review board in Gothenburg, Sweden (approval number 660-15). The projected was funded by national hospital research departments, FoU Region of Örebro Län and FoU Skaraborg and Vg Region. We have no financial or other conflicts of interest that might bias our work.

## Results

**Figure 3.**
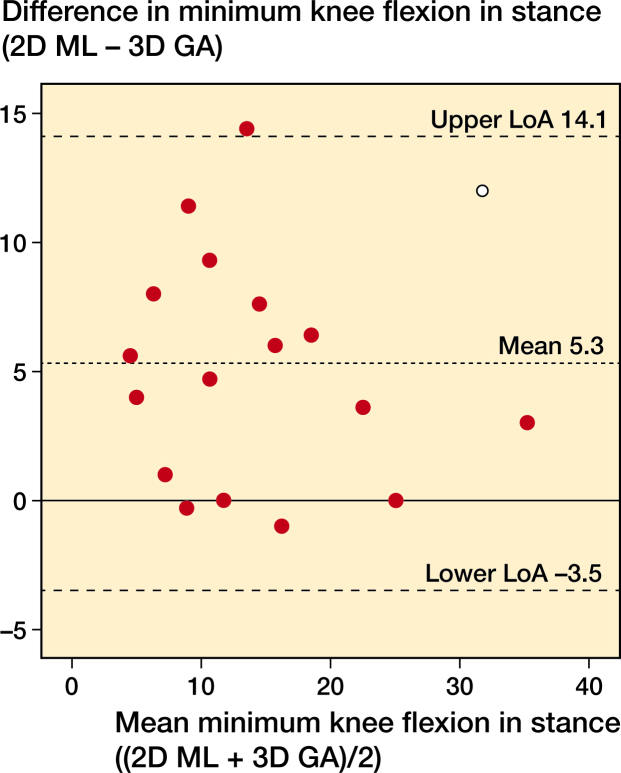
Bland–Altman plot for minimum knee flexion in stance on the left side (2D ML, 2-dimensional markerless; 3D GA, 3-dimensional gait analysis).

**Figure 4.**
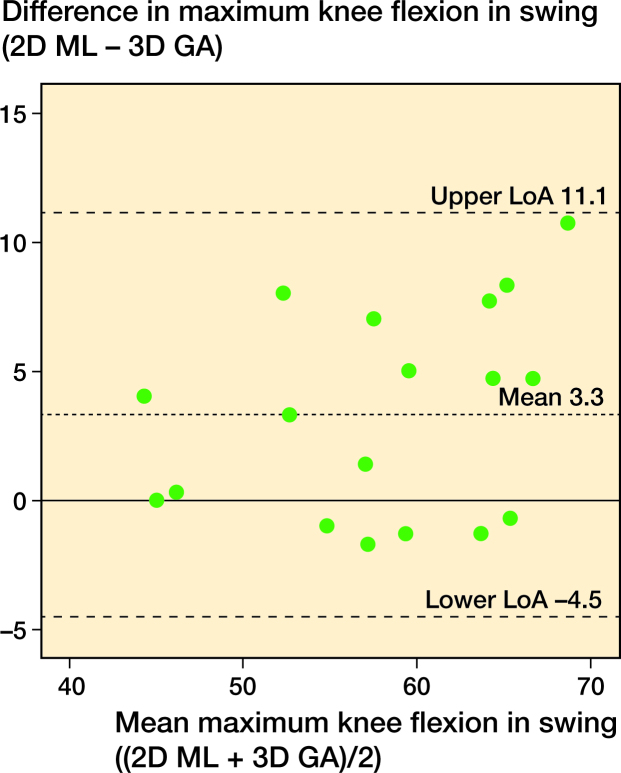
Bland–Altman plot for maximum knee flexion in swing on the left side (2D ML, 2-dimensional markerless 3D GA, 3-dimensional gait analysis).

Overall, the mean statistical differences of the gait variables as estimated by 2D ML and 3D GA (2D ML minus 3D GA) ranged from +3.3 to +7.0 degrees (CI 1.3–9.0) (Table 1). These differences remained statistically significant after Bonferroni correction except for 2 gait variables (Initial contact and Loading response, left side) that, after Bonferroni correction, were not statistically significant using the Spearman correlation coefficient. The agreement of the 2D ML and 3D GA estimates for knee extension in stance, left side, and knee flexion in swing, left side, are illustrated with Bland–Altman plots (Figures 3 and 4). The reliability of 2D ML and 3D GA is reported in Table 2. The 2D ML measurements revealed moderate to excellent reliability, with ICC CI between 0.62 and 0.98. The 3D GA measurements showed similar reliability except for the variable Loading response, where the ICC CI was 0.41–0.89, indicating poor reliability. The resulting variables exhibited moderate to excellent results in the 3D GA estimates, with ICC CI between 0.70 and 0.98 (Koo and Li 2016).

**Table 1.** Variables, side, and mean angles for the 2-dimensional markerless (2D ML) method and 3-dimensional gait analysis (3D GA)

Gait variables (°)	2D ML	3D GA	Difference				Correlation	
	and side	mean (SD)	mean (SD)	mean (95% CI)	p-value^a^	95% LoA	coefficient	p-value^b^
Knee flexion at initial contact								
	Right	26.1 (9.5)	19.1 (9.8)	7.0 (5.0–9.0)	< 0.001	–0.9 to 14.8	0.16	0.5
	Left	26.8 (10.6)	20.6 (8.5)	6.3 (4.2–8.2)	< 0.001	–1.6 to 14.1	0.47	0.05
Knee flexion at loading response								
	Right	32.4 (9.5)	26.3 (8.7)	6.1 (4.2–8.0)	< 0.001	–1.1 to 13.4	0.22	0.4
	Left	34.0 (9.3)	27.7 (7.1)	6.3 (4.2–8.2)	< 0.001	–1.6 to 14.1	0.49	0.04
Knee flexion in stance								
	Right	17.3 (11.7)	10.6 (12.5)	6.7 (4.9–8.3)	< 0.001	–0.2 to 13.5	–0.19	0.4
	Left	17.5 (9.1)	12.2 (9.1)	5.3 (3.1–7.6)	< 0.001	–3.5 to 14.1	–0.052	0.8
Knee flexion in swing								
	Right	60.3 (11.4)	55.4 (9.8)	5.0 (2.7–7.3)	< 0.001	–4.1 to 13.9	0.26	0.3
	Left	59.6 (8.4)	56.4 (7.2)	3.3 (1.3–5.2)	< 0.001	–4.5 to 11.1	0.31	0.2

Mean difference calculated by subtracting the 3D GA angle from the 2D ML angle in degrees (SD, standard deviation; CI, confidence interval; LoA, limits of agreement).

a p-value of the mean difference.

b p-value of the Spearmen correlation coefficient.

**Table 2.** Comparison of estimates from the 2-dimensional markerless (2D ML) method and 3-dimensional gait analysis (3D GA)

	Gait variables	2D ML	3D GA
	Side	ICC (95% CI)	ICC (95% CI)
Knee flexion at initial contact			
	Right	0.85 (0.67–0.94)	0.87 (0.70–0.95)
	Left	0.96 (0.92–0.96)	0.95 (0.89–0.98)
Knee flexion at loading response			
	Right	0.83 (0.62–0.93)	0.73 (0.41–0.89)
	Left	0.93 (0.84–0.97)	0.95 (0.89–0.98)
Knee flexion in stance			
	Right	0.89 (0.74–0.96)	0.88 (0.73–0.95)
	Left	0.93 (0.85–0.97)	0.97 (0.93–0.99)
Knee flexion in swing			
	Right	0.92 (0.82–0.97)	0.90 (0.78–0.96)
	Left	0.94 (0.87–0.98)	0.94 (0.87–0.98)

The 18 participants completed 3 trials for each side.

## Discussion

We found a mean difference between the 2D ML and 3D GA estimates of knee joint angles ranging from + 3.3 to +7.0 degrees. For both the 2D ML and 3D GA methods, the within-method reliability was mostly good to excellent.

The 95% LoA reveal how well the 2D ML and 3D GA methods agree. The LoA for all gait variables showed an expectedly large range (from 4.5 to 15 degrees), resulting from the high gait variability observed over the heterogeneous subject cohort analyzed. Several factors can explain the differences found in the knee estimated by 2D ML and 3D GA. First, the anatomical coordinate system definition for the femur and tibia differs between the methods. Second, we compared the knee flexion and extension angles obtained by projecting the participant movement in the sagittal plane (2D ML) with a 3D joint kinematic description based on Euler angle decomposition (3D GA). Third, 2D ML tracked the body segment movement based on the segment contours, whereas 3D GA is based on skin marker trajectories; therefore different soft tissue artefacts should be expected, and their effects on the knee kinematics should be different. However, despite the unavoidable differences found between the methods, it is important to highlight that for both 2D ML and 3D GA the within-method reliability was mostly good to excellent.

There is consensus in the literature that the knee joint is one of the more difficult to assess using the Edinburgh video Visual Gait Score reflecting a degree of agreement with the measurements obtained using a 3D GA of 47%–63% (Read et al. 2003) and 52–71% (del Pilar Duque Orozco et al. 2016). Both studies showed the usefulness of a video-based scoring system but with some limitations, as the previous experience of the observer and the training were both demonstrated to play a role in the agreement. Concurrent validity of most previous 2D ML methods has been assessed only on healthy participants (Sandau et al. 2014, Castelli et al. 2015, Saner et al. 2017). Sandau et al. (2014) applied their 2D ML method on 10 healthy adults and found a general overestimation of knee joint kinematics (mean error ± SD) equal to 2.8 ± (1.9) degrees for knee flexion and extension when compared against a 3D GA system. Castelli et al. (2015) found in 10 healthy participants an average root-mean-square difference between the knee joint angles estimated by 2D ML and 3D GA, over the entire gait cycle, of up to 4.4 degrees. In a recent study, Saner et al. (2017) proposed a method based on a low-cost webcam technology for knee sagittal kinematics estimation, which showed good to excellent reliability but lacked sufficient reliability during knee-stance. Unfortunately, no concurrent validation against a 3D GA system was performed. Furthermore, it should be noted that whereas participants in our study walked at a self-selected speed on a walkway with no constriction to arm movement, in the study reported by Saner et al. (2017) participants walked on a treadmill at a set speed and held their hands and arms up on a handrail, a setup that inevitably influences walking patterns and is difficult to apply to individuals with CP.

There is no exact definition or classification of crouch. Miller (2005) proposed 3 different intervals: mild (10–25 degrees), moderate (26–45 degrees), and severe (over 45 degrees). Others defined severe crouch as over 30 degrees of flexion in mid-stance (Rodda and Graham 2001). From a clinical point of view, we think the 2D ML assessment can prove useful, for example in stance phase. A change from mild to moderate, or moderate to severe crouch, could be detected with the 2D ML system despite overestimating range of motion with a mean of 3.3–7.0 degrees, since the within-method reliability was mostly good to excellent. Certainly, using the 2D ML system, flexion increase of 10 degrees in mid-stance could be detected and was considered a significant change motivating a full 3D GA and a clinical evaluation for possible treatment to avoid progress of crouch.

In Sweden, a systematic follow-up program for children with CP (CPUP) has been conducted since the 1990s (Hägg­lund et al. 2005). According to the 2013 yearly CPUP report, 61% of children with CP ambulate independently (GMFCS levels I and II) and 8% need support when walking (GMFCS level III) (CPUP yearly report 2013, Palisano et al. 1997). This means that ambulatory children constitute a substantial number where gait deterioration over time may be expected (Opheim et al. 2013). However, no dynamic assessment of gait in children with CP is currently included in the CPUP, and hence an objective tool to detect and follow the development of gait impairment is lacking.

It is important to highlight that the purpose of the proposed 2D ML method is not to replace 3D GA, but rather to make an additional tool available to a greater number of children with CP, which could make longitudinal follow-up possible. A case report (Butler et al. 2016) and previously, Bell et al. (2002) showed that, using 3D GA, it was possible to follow and detect changes in the gait patterns in CP over several years. 3D GA has thus shown itself to be useful in follow-up, and therefore it is reasonable to assume that 2D ML can also be used for long-term follow-up and screening.

A disadvantage with the 2D ML system is obviously the lack of frontal and rotational plane assessment, which is especially important in CP, where the rotational plane deformity often needs to be corrected with bony surgery. Nevertheless, when progression of deviation is identified with the 2D ML system, a more comprehensive assessment with 3D GA, as mentioned above, may be indicated.

The 2D ML method we propose is composed of inexpensive, commercially available equipment and requires limited hands-on for processing. Overall, when the clinical interest is limited to kinematic analysis in the sagittal plane, the 2D ML system may represent a valid alternative to traditional 3D GA by making the gait analysis low-cost and avoiding the use of skin markers. The 2D ML method offers a reliable quantification of movement. It is a simpler and quicker measurement than 3D GA but, at the same time, more valuable than the visual analysis based on manual anatomical landmark digitalization.

With the relatively subtle involvement of CP, we were unable to study whether those individuals with more severe gait deviations would show similar results. The rotational profile of the participants in this study was not assessed, which naturally could influence the results. We present, and assessed, the knee joint kinematics only, although the proposed 2D ML method can also estimate hip and ankle joint kinematics. However, a full validation of the lower limb kinematics was beyond the scope of our study.

In summary, despite overestimating, 2D ML is a convenient tool that could be used to assess knee joint angles in the sagittal plane and, more importantly, detect changes over time in a follow-up program in ambulatory children with CP.

### Supplementary data

Appendix is available as supplementary data in the online version of this article, http://dx.doi.org/10.1080/17453674.2018.1525195

EPC: Main investigator, assessment of medical records, recruitment of participants, performing statistical analysis, and writing the manuscript. AC: Organizing video assessments, interpretation of the 2D ML assessments, and writing the manuscript with special emphasis on methods. GF, GP, NV: Main responsibility for 2D ML video assessments. UDC: Participated in writing and editing the manuscript. AM: Participated in performing the statistical analysis and writing of the manuscript. JR: Planning and organization, recruitment of participants and writing the manuscript

## References

[CIT0001] Bell K J, Ounpuu S, DeLuca P A, Romness M J Natural progression of gait in children with cerebral palsy. J Pediatr Orthop 2002; 22(5): 677–82. 12198474

[CIT0002] Benedetti M G, Catani F, Leardini A, Pignotti E, Giannini S Data management in gait analysis for clinical applications. Clinical Biomechanics 1998; 13(3): 204–15. 11415789 10.1016/s0268-0033(97)00041-7

[CIT0003] Butler E E, Steele K M, Torburn L, Gamble J G, Rose J Clinical motion analyses over eight consecutive years in a child with crouch gait: a case report. J Med Case Rep 2016; 10: 157. 27301473 10.1186/s13256-016-0920-9 PMC4908800

[CIT0004] Castelli A, Paolini G, Cereatti A, Della Croce U A 2D Markerless gait analysis methodology: validation on healthy subjects. Comput Math Methods Med 2015; 2015: 186780. 26064181 10.1155/2015/186780 PMC4430646

[CIT0005] CPUP. Uppföljningsprogram för Cerebral pares: Årsrapport. 2013 Available from: http://cpup.se/wp-content/uploads/2014/03/Arsrapport2013_1.pdf.

[CIT0006] Del Pilar Duque Orozco M, Abousamra O, Church C, Lennon N, Henley J, Rogers K J, Sees J P, Connor J, Miller F Reliability and validity of Edinburgh visual gait score as an evaluation tool for children with cerebral palsy. Gait Posture 2016; 49: 14–18. 27344448 10.1016/j.gaitpost.2016.06.017

[CIT0007] DeLuca P A Gait analysis in the treatment of the ambulatory child with cerebral palsy. Clin Orthop Relat Res 1991; (264): 65–75. 1997253

[CIT0008] Eastlack M E, Arvidson J, Snyder-Mackler L, Danoff J V, McGarvey C L Interrater reliability of videotaped observational gait-analysis assessments. Phys Ther 1991; 71(6): 465–72. 2034709 10.1093/ptj/71.6.465

[CIT0009] Gage J R, DeLuca P A, Renshaw T S Gait analysis: principle and applications with emphasis on its use in cerebral palsy. Instr Course Lect 1996; 45: 491–507. 8727765

[CIT0010] Gupta S, Raja K Responsiveness of Edinburgh Visual Gait Score to orthopedic surgical intervention of the lower limbs in children with cerebral palsy. Am J Phys Med Rehabil 2012; 91(9): 761–7. 22790796 10.1097/PHM.0b013e31825f1c4d

[CIT0011] Hägglund G, Andersson S, Duppe H, Lauge-Pedersen H, Nordmark E, Westbom L Prevention of dislocation of the hip in children with cerebral palsy: the first ten years of a population-based prevention programme. J Bone Joint Surg Br 2005; 87(1): 95–101. 15686244

[CIT0012] Kawamura C M, de Morais Filho M C, Barreto M M, de Paula Asa S K, Juliano Y, Novo N F Comparison between visual and three-dimensional gait analysis in patients with spastic diplegic cerebral palsy. Gait Posture 2007; 25(1): 18–24. 16431106 10.1016/j.gaitpost.2005.12.005

[CIT0013] Koo T K, Li M Y A guideline of selecting and reporting intraclass correlation coefficients for reliability research. J Chiropr Med 2016; 15(2): 155–63. 27330520 10.1016/j.jcm.2016.02.012 PMC4913118

[CIT0014] Maathuis K G, van der Schans C P, van Iperen A, Rietman H S, Geertzen J H Gait in children with cerebral palsy: observer reliabilityof Physician Rating Scale and Edinburgh Visual Gait Analysis Interval Testing scale. J Pediatr Orthop 2005; 25(3): 268–72. 10.1097/01.bpo.0000151061.92850.74 15832135

[CIT0015] Mackey A H, Lobb G L, Walt S E, Stott N S Reliability and validity of the Observational Gait Scale in children with spastic diplegia. Dev Med Child Neurol 2003; 45(1): 4–11. 12549749

[CIT0016] Miller F Cerebral palsy [Elektronisk resurs]. New York: Springer Science + Business Media; 2005.

[CIT0017] Narayanan U G The role of gait analysis in the orthopaedic management of ambulatory cerebral palsy. Curr Opin Pediatr 2007; 19(1): 38–43. 17224660 10.1097/MOP.0b013e3280118a6d

[CIT0018] Ong A M, Hillman S J, Robb J E Reliability and validity of the Edinburgh Visual Gait Score for cerebral palsy when used by inexperienced observers. Gait Posture 2008; 28(2): 323–6. 18328710 10.1016/j.gaitpost.2008.01.008

[CIT0019] Opheim A, McGinley J L, Olsson E, Stanghelle J K, Jahnsen R Walking deterioration and gait analysis in adults with spastic bilateral cerebral palsy. Gait Posture 2013; 37(2): 165–71. 22818116 10.1016/j.gaitpost.2012.06.032

[CIT0020] Palisano R, Rosenbaum P, Walter S, Russell D, Wood E, Galuppi B Development and reliability of a system to classify gross motor function in children with cerebral palsy. Dev Med Child Neurol 1997; 39(4): 214–23. 10.1111/j.1469-8749.1997.tb07414.x 9183258

[CIT0021] Perry J Gait analysis, normal and pathological function. Thorofare, NJ: SLACK; 1992.

[CIT0022] Rathinam C, Bateman A, Peirson J, Skinner J Observational gait assessment tools in paediatrics: a systematic review. Gait Posture 2014; 40(2): 279–85. 24798609 10.1016/j.gaitpost.2014.04.187

[CIT0023] Read H S, Hazlewood M E, Hillman S J, Prescott R J, Robb J E Edinburgh visual gait score for use in cerebral palsy. J Pediatr Orthop 2003; 23(3): 296–301. 12724590

[CIT0024] Rodda J, Graham H K Classification of gait patterns in spastic hemiplegia and spastic diplegia: a basis for a management algorithm. Eur J Neurol 2001; 8 (Suppl 5): 98–108. 11851738 10.1046/j.1468-1331.2001.00042.x

[CIT0025] Rodda J M, Graham H K, Carson L, Galea M P, Wolfe R Sagittal gait patterns in spastic diplegia. J Bone Joint Surg Br 2004; 86(2): 251–8. 15046442 10.1302/0301-620x.86b2.13878

[CIT0026] Sandau M, Koblauch H, Moeslund T B, Aanaes H, Alkjaer T, Simonsen E B Markerless motion capture can provide reliable 3D gait kinematics in the sagittal and frontal plane. Med Eng Phys 2014; 36(9): 1168–75. 25085672 10.1016/j.medengphy.2014.07.007

[CIT0027] Saner R J, Washabaugh E P, Krishnan C Reliable sagittal plane kinematic gait assessments are feasible using low-cost webcam technology. Gait Posture 2017; 56: 19–23. 28482201 10.1016/j.gaitpost.2017.04.030 PMC5515224

[CIT0028] Sangeux M, Armand S Kinematic deviations in children with cerebral palsy In: Orthopedic management of children with cerebral palsy: a comprehensive approach. Hauppauge, NY: Nova Science Publishers; 2015.

[CIT0029] Surer E, Cereatti A, Grosso E, Della Croce U A markerless estimation of the ankle-foot complex 2D kinematics during stance. Gait Posture 2011; 33(4): 532–7. 21295984 10.1016/j.gaitpost.2011.01.003

[CIT0030] Ugbolue U C, Papi E, Kaliarntas K T, Kerr A, Earl L, Pomeroy V M, Rowe P J The evaluation of an inexpensive, 2D, video based gait assessment system for clinical use. Gait Posture 2013; 38(3): 483–9. 23465758 10.1016/j.gaitpost.2013.01.018

